# Simultaneously Mitigation of Acrylamide, 5-Hydroxymethylfurfural, and Oil Content in Fried Dough Twist via Different Ingredients Combination and Infrared-Assisted Deep-Frying

**DOI:** 10.3390/foods10030604

**Published:** 2021-03-12

**Authors:** Zhonghui Han, Jianxin Gao, Shunyang Zhang, Yan Zhang, Shuo Wang

**Affiliations:** 1 Tianjin Key Laboratory of Food Science and Health, School of Medicine, Nankai University, Tianjin 300071, China; zhhan@qlu.edu.cn (Z.H.); wangshuo@nankai.edu.cn (S.W.); 2 College of Food Science and Engineering, Qilu University of Technology (Shandong Academy of Sciences), Jinan 250353, China; 3 College of Life Science, Shandong Normal University, Jinan 250014, China; 618160@sdnu.edu.cn; 4 College of Food Science and Engineering, Tianjin University of Science and Technology, Tianjin 300457, China; jhzhang@mail.tust.edu.cn

**Keywords:** fried dough twist, acrylamide, 5-hydroxymethylfurfural, oil content

## Abstract

The effect of main ingredients (wheat flours, polyol sweeteners, and frying oil) and infrared-assisted deep-frying on the acrylamide, 5-hydroxymethylfurfural (HMF), oil content, and physicochemical characteristics of fried dough twist (FDT) were investigated. The amount of acrylamide and HMF produced in FDT made with low-gluten flour is significantly lower than that of flour with high gluten content. Among polyol sweeteners, maltitol causes the greatest reduction in acrylamide and HMF in FDT. Moreover, the oil content of FDT was significantly reduced by optimizing the infrared-assisted deep-frying process. At last, compared with deep-frying FDT made of sucrose, infrared-assisted deep-frying FDT made of maltitol reduced acrylamide, HMF, and oil content by 61.8%, 63.4%, and 27.5%, respectively. This study clearly showed that the ingredients, flour and polyol sweeteners used to process FDT are the two major determinants of the formation of acrylamide and HMF in FDT, and infrared-assisted deep-frying can significantly affect the oil content in FDT. Simultaneously, the mitigation of the acrylamide, HMF, and oil content in FDT can be achieved by using low-gluten flour and maltitol in the ingredients, combined with infrared-assisted deep-frying.

## 1. Introduction

Fried dough twist (FDT) is a traditional Chinese fried food, which is made by mixing wheat flour, sucrose, and other ingredients [1,2]. It is popular among consumers in China, North Korea, South Korea, and other countries, mainly due to FDT’s unique color, flavor, and texture formed by the Maillard reaction. 

The Maillard reaction that occurs between the amino acids (or proteins) and the reducing sugar during the heating process is critical to the formation of color, flavor, and taste. For instance, the melanoidins formed by Maillard reaction can promote the formation of attractive colors in food, and the formed pyrazine and furan have a pleasant nutty taste and caramel aroma in food [3,4]. The Maillard reaction can also induce the formation of high molecular weight polymers by affecting the structure of the proteins, resulting in the increase in hardness of protein bars [5,6].

**Figure 1:**
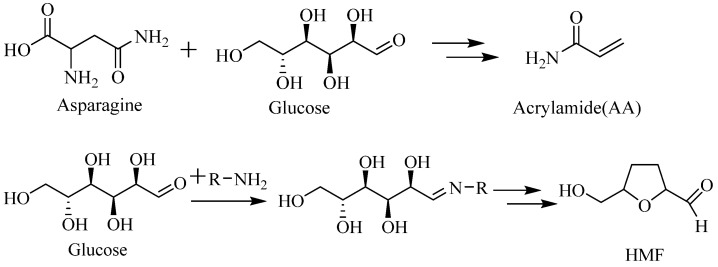
The formation of acrylamide (AA) and hydroxymethylfurfural (HMF).

Nevertheless, the Maillard reaction can also promote the formation of potentially harmful Maillard reaction products (MRPs) such as acrylamide and 5-hydroxymethylfurfural (HMF) in foods [1,2,7,8,9]. Acrylamide in food is mainly generated by the reaction of asparagine with reducing sugars (glucose and fructose) as precursors (Figure 1) [10]. Many studies have demonstrated that acrylamide has genotoxicity, neurotoxicity, and carcinogenicity effects on human cells and animals [11,12]. HMF is mainly formed by the Amadori rearrangement product of hexose or by the direct dehydration of sugars when the pH value is equal to or less than 7 (Figure 1) [13,14]. HMF has also been confirmed to have genotoxicity, neurotoxicity, and carcinogenicity effects on human cells and animals [11,12]. HMF was confirmed to have carcinogenicity and genotoxicity in animals and human cells [8,15]. Acrylamide and HMF have been detected in many food products, especially in high-temperature processing foods such as frying (fried potato products), baking (biscuits and bread), and roasting (roasted coffee) [9,12,16,17]. The content of acrylamide in heated foodstuffs can reach up to 4804 μg kg^−1^ [18]. To reduce the risk of potentially harmful substances that possibly cause adverse effects on human health, researchers have been actively seeking approaches to different ways of processing foodstuffs. For example, reducing asparagine in potatoes can reduce the formation of acrylamide in French fries, and adding additives can reduce the formation of acrylamide and HMF in the biscuit [19,20,21].

Besides, deep-fried FDT contains a lot of fried oil. Studies have shown that an excessive intake of high-fat foods can cause a variety of diseases, such as obesity, cardiovascular, and Alzheimer’s disease. Therefore, it is an inevitable development to reduce the oil content in FDT to meet consumer demand for healthy foods [22]. There are many ways to reduce the oil content in fried foods, many of which are based on the relationship between food moisture content and oil content during the frying process. The chickpea flour-based snack food and potato slices can be pretreated by an infrared, oven, and other different methods to reduce the moisture content and the water-oil transfer gradient during the frying process, thereby reducing the oil content of the food [23,24].

As mentioned above, many factors affect physicochemical characteristics, potentially harmful MRPs, and oil content in foods, including recipe ingredients, processing methods, processing temperature (time), and so on. However, little is known about the effect of the main ingredients and processing methods on potentially harmful MRPs, oil content, as well as related physicochemical characteristics of FDT during the manufacturing process. Therefore, the main objectives of this study were to (i) evaluate the effects of wheat flours, polyol sweeteners, frying oil on acrylamide, HMF, oil content, and physicochemical characteristics during the manufacturing process of FDT, and (ii) to develop a strategy of infrared-assisted deep-frying to reduce the formation of unhealthy MRPs and oil content for enhancing the safety and quality of FDT.

## 2. Materials and Methods

### 2.1. Materials

Standards of 5-hydroxymethylfurfural (HMF), acrylamide, and *d*_3_-acrylamide were obtained from Sigma-Aldrich (St.Louis, MO, USA). Zinc acetate, petroleum ether, formic acid, potassium hexacyanoferrate, n-hexane, petroleum ether, acetonitrile (HPLC grade), and methanol were purchased from J&K Scientific (Beijing, China).

Wheat flour (low-gluten flour, medium-gluten flour, and strong-gluten flour) were purchased from Weifang Kite Flour Co., Ltd. (Weifang, Shangdong, China). Yeast was provided by Angel Yeast Co., Ltd. (Yichang, Hubei, China). Sucrose was purchased from Guangzhou Fuzheng Donghai Food Co., Ltd. (Guangzhou, Guangdong, China). Maltitol, sorbitol, and lactitol were provided by Shandong Green Construction Biotechnology Co., Ltd. (Dezhou, Shangdong, China). Palm oil, soybean oil, and rapeseed oil were purchased from COFCO Corporation (Beijing, China). Shortening was obtained from Shanghai Gaofu Longhui Food Co., Ltd. (Shanghai, China).

### 2.2. Preparation of FDT

The formula of FDT is provided by the food manufacturer with minor modifications. Ingredients and their weight proportions were 100 g of wheat flour, 10 g of sweeteners, 1 g of dry yeast, 5 g of shortening, and 40 mL of water. Firstly, the dough was kneaded, and 1 g of yeast and 2 g of sweetener was mixed into 40 mL of water (temperature 40 °C) to form a solution, then the solution was slowly added to 100 g of flour and continuously stirred for 2 min, followed by 5 g of shortening added, and then kneaded repeatedly until the dough surface was smooth. The dough was rested in a proofing cabinet (37 °C, 85% RH) for 30 min. For shaping, 8 g of sucrose was added to the dough and kneaded for 4 min, then kneaded into a cylindrical strip with a diameter of 1 cm. The dough was then divided by sections and cut by 15 cm, folded in half, and twisted into a rope shape. Finally, the shaped-dough was fried with oil at 160 °C (or infrared equipment pretreatment (Meibo, Zhenjiang, China) deep-frying). 

### 2.3. Quantification of Acrylamide in FDT

Acrylamide of the sample was determined according to a previously performed method [25], with minor modifications. 

A total of 10 mg/L of *d_3_*-acrylamide internal standard solution (10 μL) and deionized water (9 mL) were added to 15 mL plastic centrifuge tubes containing 1.0 g of FDT powder, followed by 0.5 mL of Carrez I and II reagent. The mixed solutions were shaken at 40 °C for 30 min, and then centrifuged at 5000× *g* for 10 min, finally, the supernatant was taken for purification. The n-hexane (10 mL) was added to the supernatant and shaken for 10 min, and then removed, followed by purification of the acrylamide using a pretreated Waters Oasis HLB SPE column (200 mg, 30 μm). The prepared sample was centrifuged at 8000× *g* for 10 min to obtain the supernatant, which was then filtered by a 0.22 μm nylon filter and used for liquid chromatography-mass spectrometry (LC-MS/MS) analysis.

LC-MS/MS analysis was performed on an Agilent 1200 LC system (Agilent, Waldbronn, Germany) connected to an Agilent 6410 triple-quadrupole mass spectrometer (Agilent, Waldbronn, Germany) in ESI+ and MRM mode. The nebulizer, drying, and collision gas was nitrogen. The nebulizer pressure, drying gas flow rate, and capillary voltage were 40 psi, 10 L·min^−1^ (350 °C), and 4 kV, respectively. The analytical column was an Agilent ZORBAX Eclipse on a C-18 column (2.1 × 150 mm, 3.5 μm) maintained at 35 °C. The mobile phase was methanol:water (5:95, *v/v*) mixture with a flow rate of 0.2 mL·min^−1^, running for 15 min. The injection volume was 2 μL. *m/z* 72.2→*m/z* 55.4 and *m/z* 75.3→*m/z* 58.4 transitions for acrylamide and *d_3_*-acrylamide, respectively. The limit of detection (LOD) in samples was 6 μg·kg^−1^ and the limit of quantification (LOQ) was 16 μg·kg^−1^. 

### 2.4. Quantification of HMF in FDT

The analysis of HMF was carried out according to our previously established method [25,26], with minor modifications. For a sample of 1 g of FDT powder in plastic centrifuge tubes, 8 mL of deionized water and 0.5 mL of Carrez I and II solutions were added. The mixtures were vortexed for 3 min and then shaken for 30 min at 40 °C, subsequently, centrifuged at 5000× *g* for 10 min, to obtain clear supernatants. The HMF in supernatants was purified by applying the Waters Oasis HLB SPE cartridges (200 mg, 30 μm). The prepared samples were filtered by a 0.22 μm nylon filter, and then analyzed with HPLC.

Instrumental analysis was performed using a Shimadzu HPLC system (Shimadzu, Kyoto, Japan) consisting of a CBM-20A system controller, two LC-20AT pumps, a DGU-20A degasser, an LC-20A UV detector, and a CTO-20AT column oven. The analysis system was equipped with a Thermal ODS C18 analysis column (4.6 mm × 250 mm, 5 μm). The temperature of the column oven was set to 35 °C. The UV detection wavelengths was 284 nm. The mobile phase was acetonitrile:water (5:95, *v/v*) at a flow rate of 0.6 mL·min^−1^, and the run time was 20 min per sample. The injection volume was 20 μL. LOD and LOQ of the samples were 0.06 mg·kg^−1^ and 0.15 mg·kg^−1^, respectively. 

### 2.5. Analysis of Oil Content

The soxhlet extraction method was used for the determination of oil content in the sample.

### 2.6. Analysis of Moisture Content and Water Activity

The moisture content of the sample (3 g) was determined by oven drying at 105 °C for 1 h under atmospheric pressure. The water activity (a_w_) of the sample (5 g) was measured using an a_w_ instrument (LabSwift-aw, Novasina, Switzerland). Each measurement was repeated three times.

### 2.7. Analysis of FDT Color

The color measurements of FDT were carried out with a Konica Minolta CM-5 spectrocolorimeter (Tokyo, Japan). The values were recorded according to three independent parameters of CIE-L* (lightness, from 0 = black to 100 = white), a* (+a* = redness, −a* = greenness), and b* (+b* = yellowness, −b* = blueness). Each sample was analyzed six times. *Δ*E* index was calculated according to Equation (1), which allows for evaluating the total color changes in the samples. The browning index (BI) was calculated using parameters of CIE-L*, a*, b*, according to Equations (2) and (3) [27,28].

(1) $$\Delta E*=\sqrt{{\Delta a*}^{2}+{\Delta b*}^{2}+{\Delta L*}^{2}}$$

(2) BI=[100(x−0.31)]/0.17

(3) where x=(a*+1.75L*)/(5.645L*+a*−0.3012b*)

### 2.8. Analysis of FDT Texture

Texture characterization of FDT was carried out at room temperature by using a texture meter TA-XTplus (Stable Micro Systems, Surrey, UK), applying a three-point bending probe (HDP/3PB) for the test sample. The probe pre-test speed and test speed were set at 1.0 mm·s^−1^, the posttest speed was set at 10 mm·s^−1^, the probe depression distance was set to 12 mm, and the trigger force was 5 g. The maximum peak value during compression was recorded as hardness and the first peak value during compression was recorded as fracturability.

### 2.9. Statistical Analysis

Data were expressed as the mean ± standard deviation (SD) for three samples from each raw material, except for special annotations. Experimental data was performed in the analysis of variance (one-way ANOVA) by the Duncan test (*p* < 0.05). All statistical treatments were carried out using SPSS for Windows version 19.0 (SPSS Inc., Chicago, IL, USA).

## 3. Results and Discussion

### 3.1. Effect of Main Ingredients on Acrylamide and HMF in Fried Dough Twist (FDT)

**Figure 2:**
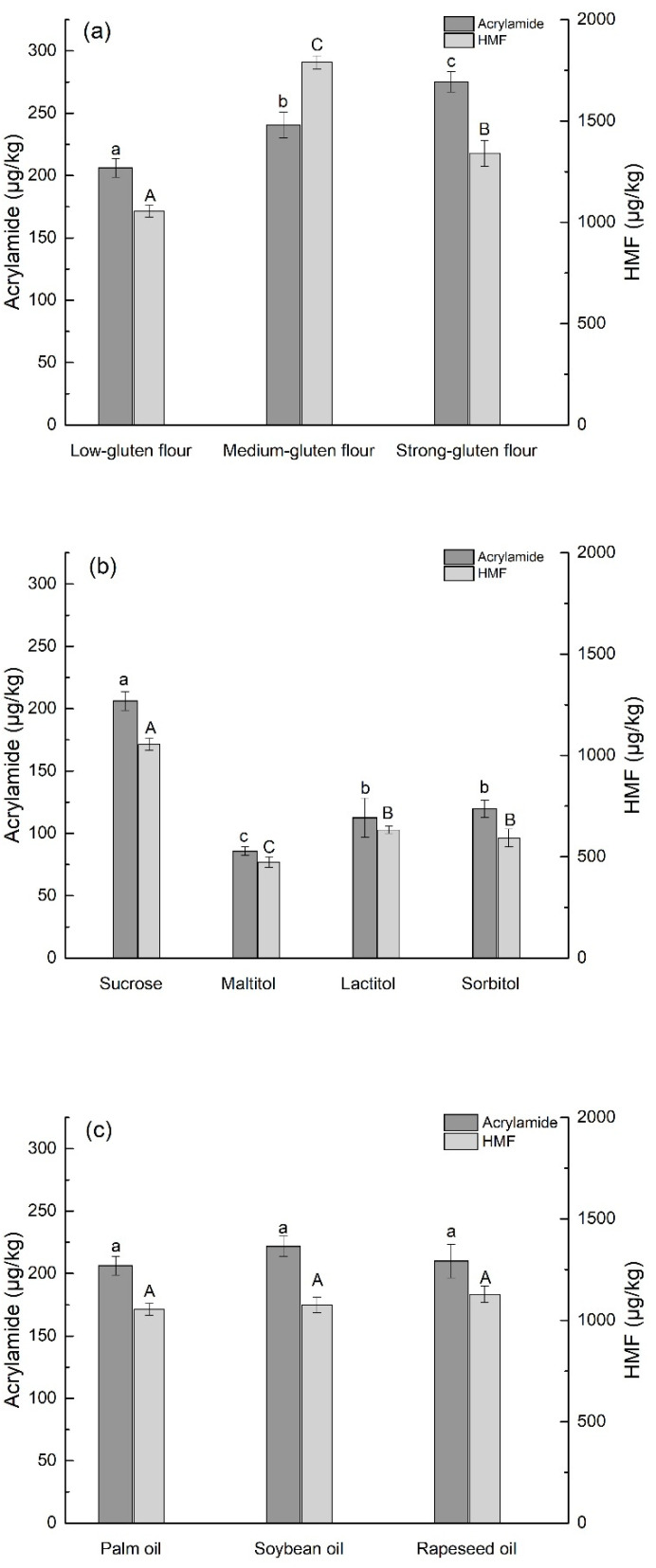
Effect of different ingredients (wheat flours (**a**), polyol sweeteners (**b**), and frying oil (**c**)) on acrylamide and 5-hydroxymethylfurfural (HMF) in fried dough twist (FDT). The different lowercase and uppercase letters represent significant difference (*p* < 0.05).

FDT was formulated with the same recipes except for flour to evaluate the effect on the formation of acrylamide and HMF. The effect of flour with different gluten content on the content of acrylamide and HMF in FDT is shown (Figure 2a). The content of acrylamide in FDT processed by strong-gluten flour was the highest (*p* < 0.05), which is 275.22 ± 8.15 μg/kg, while the highest content of HMF (1789.95 ± 32.42 μg/kg) in FDT was processed by medium strong-gluten flour. The increase in acrylamide may be ascribed to elevated levels of protein, which is similar to the previous report that crude protein may increase the acrylamide content in yeast-fermented bread [29]. Besides, the content of HMF is about several times that of acrylamide, which indicated that HMF was more easily generated than acrylamide in the thermal processing of FDT. 

The influence of different polyol sweeteners instead of sucrose on the formation of acrylamide and HMF in FDT is shown in (Figure 2b). Compared with sucrose, the contents of acrylamide and HMF in FDT with different polyol sweeteners (maltitol, lactitol, and sorbitol) was significantly reduced (*p* < 0.05). The main reason may be that the polyol sweeteners in the dough cannot be decomposed or degraded into reducing sugars, so that it cannot participate in the Maillard reaction to form potentially harmful MRPs [9,30]. The amount of acrylamide and HMF formed in FDT with maltitol was the lowest, and the contents were 85.68 ± 13.38 and 474.08 ± 26.16 μg/kg, respectively. These results suggested that the addition of polyol sweeteners instead of sugar is an effective way to mitigate potentially harmful MRPs. The effect of soybean oil and rapeseed oil on potentially harmful MRPs in FDT is shown in (Figure 2c). The results revealed that different types of frying oil have no significant effect on the formation of acrylamide and HMF in FDT. The above results indicated that the use of low-gluten flour and maltitol in the ingredients made of FDT can significantly reduce the formation of acrylamide and HMF.

### 3.2. Effect of Main Ingredients on Physicochemical of Fried Dough Twist (FDT) 

In the present study, samples of different formulas were prepared to investigate the effects of ingredients on the physicochemical characteristic analysis of the FDT. The values of the influence of wheat flours, polyol sweeteners, and frying oil on the moisture content, water activity (a_w_), color, and texture profile in the FDT are presented in Table 1.

The data showed that the moisture content of FDT made with low-gluten flour was significantly higher than that of FDT made with strong-gluten flour (*p* < 0.05). The possible reason is that the increase of surface voids in strong-gluten flour FDT dough, which makes the contact area between FDT and frying oil increase, resulting in more water evaporation and reducing the moisture content in the FDT. While the results revealed that different gluten flours had no significant effect on a_w_ of FDT, and demonstrated that FDT has low free water content and good storage properties [31]. Polyol sweeteners added had no significant effect on moisture content and a_w_ of FDT compared with the addition of sucrose. Our observation is inconsistent with previous reports. Zoulias et al. showed that the addition of polyol sweeteners in cookies has a higher moisture content and a_w_ value than the addition of sucrose [32]. This may be due to the oil more easily penetrating the food for heat transfer, which greatly reduces the moisture retention properties of polyol sweeteners during frying. Palm oil, soybean oil, and rapeseed oil were used in the fried dough respectively, and the results showed that they have no significant difference in the moisture content and a_w_ in FDT.

The values of the color parameters (a*, b*, L*, *Δ*E*, and browning index (BI)) of different formulas FDT are presented in (Table 1). Data revealed that the different gluten flour affected the value of b*, increasing from 29.05 ± 1.05 to 31.24 ± 0.65, which indicated that strong-gluten flour increased the color yellowness of FDT, but other chroma did not change significantly. BI increased from 109.00 ± 1.64 to 124.82 ± 0.97 with the gluten content in wheat flour. This observation indicated that more protein participated in the chemical reaction of FDT to promote the browning during the production process. The reason for FDT browning is similar to the browning of flour dough result of the Maillard reactions and caramelization of the exposure to high temperatures [32,33]. The b*, L*, and *Δ*E* values of FDT with maltitol, lactitol, and sorbitol added were significantly lower than that of sucrose, which was probably because polyol sweeteners are non-reducing substances and hence do not undergo Maillard browning reactions [32]. Compared with palm oil, soybean oil and rapeseed oil have no significant effect on the color change of FDT.

**Figure 3:**
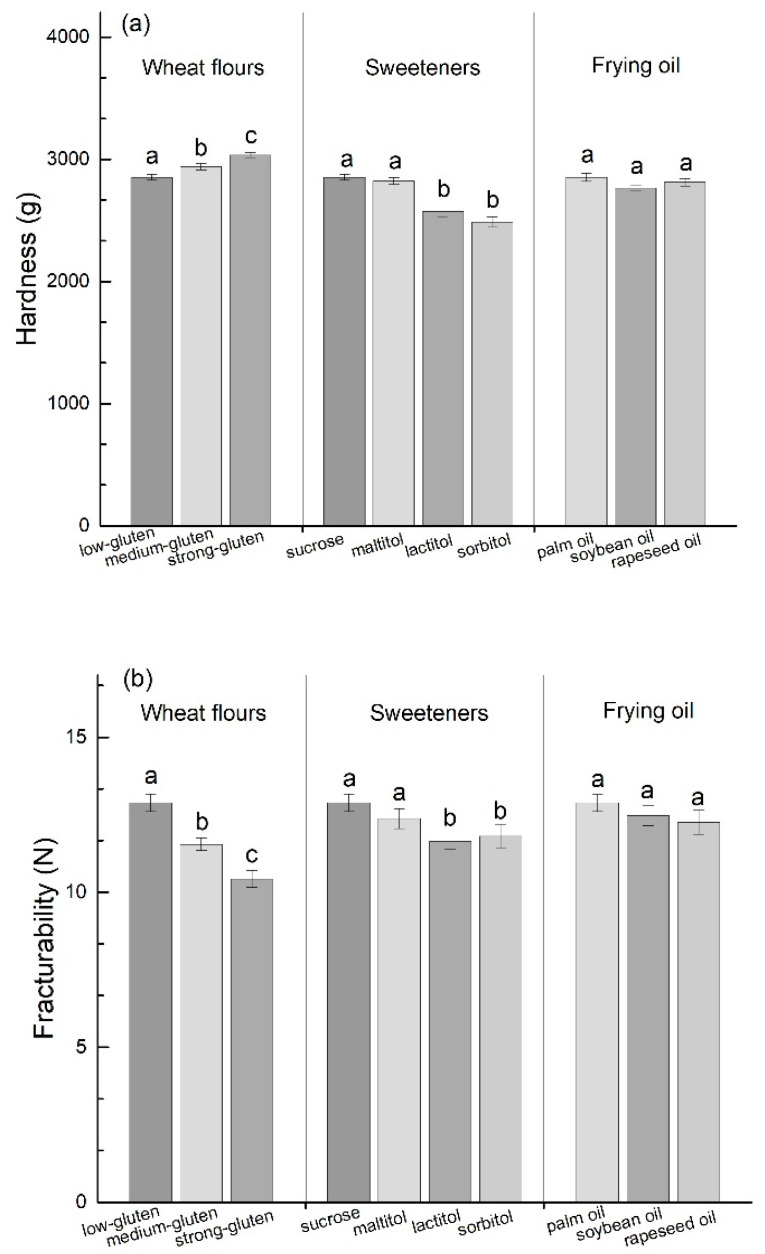
Effect of different ingredients (wheat flours, polyol sweeteners, and frying oil) on hardness (**a**) and fracturability (**b**) of fried dough twist (FDT). The different lowercase letters represent significant difference (*p* < 0.05).

Hardness and fracturability are important texture properties of FDT, according to the results obtained from the breaking test shown in (Figure 3). The hardness values of the FDT samples increased with the increase in the gluten content of the flour. resulting in the more intensive development of the gluten network, thereby promoting the hardness increase [34,35]. In addition, Graeme Baxter et al. found that the gluten content of flour positively correlated with the hardness properties of the starch gel [36], which indicated that gluten promotes the increase in hardness. However, the fracturability change of FDT is different from the hardness as it decreases with the increase of the gluten content of flour. Different polyol sweeteners also significantly affect the hardness and fracturability of FDT. FDT made with lactitol and sorbitol had lower hardness and fracturability than FDT made with sucrose. While the hardness and fracturability value of FDT made with maltitol is similar to that of FDT made with sucrose. This observation is similar to previous reports on the effect of polyol sweeteners on the hardness and fracturability of cookies [37]. Besides, the hardness and fracturability of the FDT are non-significantly influenced by soybean oil and rapeseed oil. 

The above results suggest that the content of gluten and polyol sweeteners plays a certain role in changing the physicochemical properties of FDT.

### 3.3. Effect Infrared-Assisted Deep-Frying on Fried Dough Twist (FDT)

Different wheat flours, polyol sweeteners, and frying oil have no significant effect on the oil content of the FDT (Table 1). This indicates that it is difficult to change the main ingredients to reduce the oil content in FDT. Previous studies have shown that pretreatment by using infrared can reduce the oil content of potato chips and chickpea flour-based snack food during deep-frying [22,24]. Therefore, in order to achieve the purpose of controlling the oil content in FDT, we conducted further research on infrared pretreatment combined with deep-frying. According to the above experimental results, low-gluten flour and maltitol were selected as FDT ingredients.

**Figure 4:**
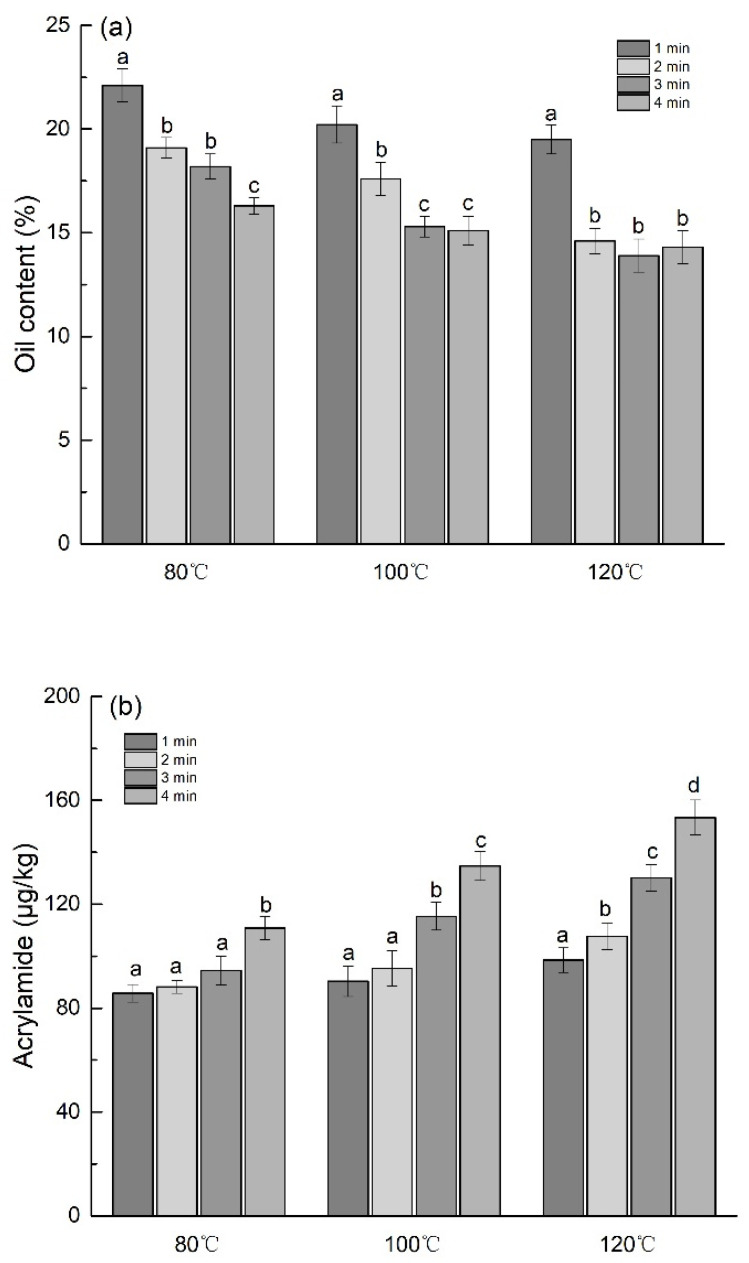

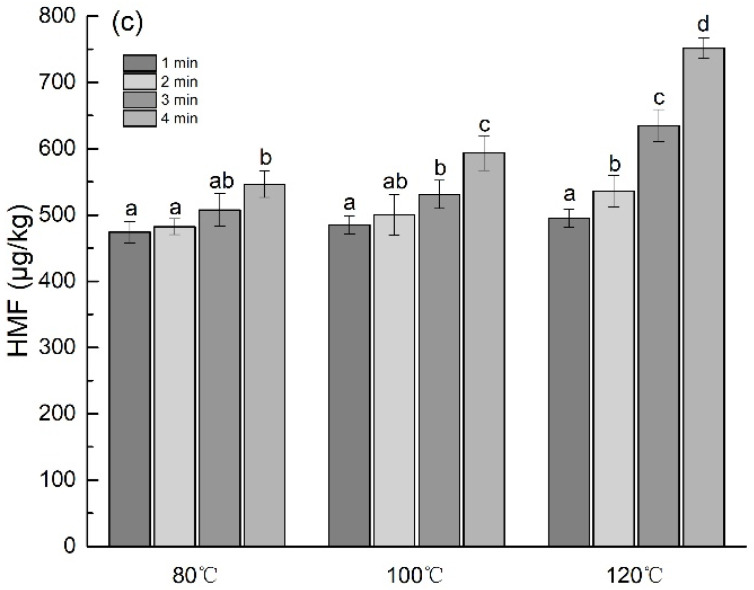
Effect of infrared pretreatment temperature and time on oil content (**a**), acrylamide (**b**), and 5-hydroxymethylfurfural (HMF) (**c**) in FDT. The different lowercase letters represent significant difference (*p* < 0.05).

The FDT dough was pretreated by infrared-assisted at different temperatures (80 °C, 100 °C, and 120 °C) and time (1 min, 2 min, 3 min, and 4 min), and then deep-fried at 160 °C for 5 min. The results of the oil content in the FDT are shown in (Figure 4a). The data revealed that the oil content in FDT decreases with the increase of infrared-assisted pretreatment temperature and time. When the condition of infrared-assisted pretreatment FDT dough is more than 3 min at 100 °C or more than 2 min at 120 °C, the oil content in FDT no longer decreases significantly. These results confirmed that infrared-assisted pretreatment can significantly reduce the oil content in the FDT under certain conditions. Based on the above experimental data, 100 °C for 3 min or 120 °C for 2 min can be preliminarily selected as infrared-assisted pretreatment conditions. In addition, the content changes of acrylamide and 5-HMF in the FDT after different infrared-assisted pretreatments were also analyzed (Figure 4b,c). The data indicated that the formation of acrylamide and 5-HMF in FDT increased with the increase of the pretreatment temperature (or time) of infrared-assisted pretreatment.

However, after infrared-assisted pretreatment, the moisture content of FDT decreased with the pretreatment temperature (or time), while the BI, hardness, and fracturability increased significantly (Table 2). This indicated that the infrared-assisted pretreatment combined with the deep-frying process affects the product’s organoleptic quality. 

To maintain the original organoleptic properties of FDT, based on the preliminary selection of infrared-assisted pretreatment conditions, the effect of deep-frying time on FDT organoleptic properties was further studied. As shown in (Table 3), the data show that the color parameters, hardness, and fracturability of FDT were increased with the increase of deep-frying time. Comparing these data with the deep-frying FDT, it is found that the organoleptic properties of FDT made by infrared-assisted pretreatment (condition at 100 °C for 3 min) combined with deep-frying (condition at 160 °C for 4 min) are more similar to that of FDT. This combined condition maintains the organoleptic properties of FDT to a certain extent.

### 3.4. Comparison of the Effect of Deep-Frying and Infrared-Assisted Deep-Frying on Fried Dough Twist (FDT)

The effect of infrared-assisted deep-frying on FDT was studied by comparing different processes on the physicochemical characteristics, oil content, and potentially harmful MRPs of FDT (Table 4). The results indicated that there were significant differences in the moisture content, color, hardness, and fracturability between deep-frying FDT with sucrose and infrared-assisted deep-frying FDT with maltitol. Nevertheless, the BI, hardness, and fracturability of FDT only differ by about 6%, 10%, and 2%, respectively. Compared with deep-frying FDT made of sucrose, the amount of acrylamide and HMF in the infrared-assisted deep-frying FDT made with maltitol were reduced by 61.8% and 63.4%, respectively. Interestingly, the content of acrylamide and HMF in the infrared-assisted deep -frying FDT made of maltitol is also lower than that in deep-frying FDT made of maltitol. This must be closely related to shortening the deep-frying time. A total of 27.5% of oil content was reduced in the infrared-assisted deep-frying FDT made of maltitol compared to that in the deep-frying FDT made of sucrose. It can be seen that changing to low-gluten flour and maltitol as ingredients, combined with infrared-assisted deep-frying process can significantly reduce the acrylamide, HMF, and oil content in FDT at the same time. 

## 4. Conclusions

Our results suggest that the formation of acrylamide and HMF in FDT made from low-gluten flour and maltitol was significantly reduced. Additionally, it also affected the water content, color, and texture characteristics of FDT, but did not significantly affect the oil content of FDT. We further researched and constructed the processing technology of combining infrared-assisted pretreatment with deep-frying. The oil content in FDT was significantly reduced by the infrared-assisted deep-frying process. Although it is shown that infrared pretreatment could increase the formation of acrylamide and HMF, the processing technology shortened the deep-frying time, resulting in a further reduction in the content of acrylamide and HMF. From these results, it can be concluded that using low-gluten flour and maltitol in the product ingredients, and combining infrared-assisted technology can be used as an effective strategy to simultaneously control acrylamide, HMF, and oil content. Further researches should explore whether consumers perceive sensory quality differences and study the impact of this practice on product shelf life.

## Figures and Tables

**Table 1:** Effect of main ingredients (wheat flours, polyol sweeteners, and frying oil) on moisture content, water activity (a_w_), color parameters, and oil content of fried dough twist (FDT).

	Wheat Flours			Polyol Sweeteners				Frying Oil		
	Low-Gluten Flour	Medium-Gluten Flour	Strong-Gluten Flour	Sucrose	Maltitol	Lactitol	Sorbitol	Palm Oil	Soybean Oil	Rapeseed Oil
Moisture content (%)	3.05 ± 0.26a	2.88 ± 0.17ab	2.56 ± 0.20b	3.05 ± 0.26a	3.18 ± 0.23a	3.25 ± 0.24a	3.03 ± 0.25a	3.05 ± 0.26a	2.92 ± 0.27a	2.88 ± 0.19a
Water activity (a_w_)	0.61 ± 0.01a	0.64 ± 0.03a	0.61 ± 0.03a	0.61 ± 0.01a	0.64 ± 0.02a	0.59 ± 0.03a	0.63 ± 0.01a	0.61 ± 0.01a	0.62 ± 0.02a	0.60 ± 0.02a
a *	16.37 ± 0.14a	15.81 ± 0.64a	15.69 ± 0.51a	16.37 ± 0.14a	15.31 ± 1.18a	15.43 ± 1.04a	16.69 ± 0.42a	16.37 ± 0.14a	16.91 ± 1.22a	14.87 ± 0.72a
b *	29.05 ± 1.05c	31.84 ± 0.42b	32.74 ± 0.65a	29.05 ± 2.45a	20.41 ± 2.26b	22.26 ± 1.79b	20.97 ± 1.42b	29.05 ± 2.45a	30.30 ± 1.58a	32.59 ± 0.85a
L *	35.69 ± 3.16a	32.71 ± 0.81a	32.41 ± 0.54a	35.69 ± 3.16a	26.78 ± 2.14b	29.50 ± 3.72b	27.49 ± 0.52b	35.69 ± 3.16a	34.15 ± 3.15a	37.59 ± 1.31a
*Δ*E *	-	4.12 ± 0.64b	4.98 ± 1.07b	-	12.45 ± 1.23a	9.27 ± 2.06a	11.52 ± 1.43a	-	2.06 ± 1.16b	4.29 ± 1.28b
BI	109.00 ± 1.64c	122.08 ± 1.19b	124.82 ± 0.97a	109.00 ± 1.64a	106.72 ± 1.48b	105.17 ± 1.05b	107.43 ± 1.69ab	109.00 ± 1.64a	116.69 ± 1.70a	113.75 ± 1.01a
Oil content (%)	21.1 ± 1.4a	19.3 ± 1.8a	20.6 ± 1.5a	21.1 ± 1.4a	20.6 ± 0.8a	19.6 ± 1.5a	21.9 ± 1.4a	21.1 ± 1.4a	20.1 ± 1.1a	21.5 ± 1.5a

1 Data are presented as mean values ± standard deviation. 2 The different lowercase letters represents significant difference in the same column (* *p* < 0.05).

**Table 2:** Effect of infrared-assisted temperature and deep-frying time on moisture content, water activity (a_w_), color parameters, hardness, and fracturability of fried dough twist (FDT).

Infrared-Assisted Temperature	Infrared-Assisted Time (min)	Moisture Content (%)	Water Activity	a *	b *	L *	*Δ*E *	BI	Hardness(g)	Fracturability (N)
80 °C	1	3.11 ± 0.25a	0.64 ± 0.03a	15.64 ± 1.13a	20.41 ± 0.97a	41.47 ± 0.29a	-	84.45 ± 2.28a	2914.25 ± 60.74a	12.56 ± 0.72a
	2	3.02 ± 0.32ab	0.58 ± 0.02a	14.80 ± 1.08a	24.66 ± 1.75b	38.20 ± 0.55b	5.43 ± 1.23b	94.23 ± 1.37b	3325.73 ± 38.19b	12.45 ± 0.79a
	3	2.85 ± 0.28ab	0.59 ± 0.01a	14.65 ± 0.56a	27.92 ± 2.32c	35.76 ± 0.62c	9.48 ± 1.43c	104.81 ± 2.35c	3484.33 ± 45.28b	13.15 ± 0.35ab
	4	2.62 ± 0.24b	058 ± 0.03a	13.65 ± 2.60a	29.72 ± 1.28d	33.21 ± 0.78c	12.60 ± 0.95d	115.89 ± 1.46d	3633.84 ± 61.32b	14.03 ± 0.27b
100 °C	1	2.93 ± 0.23a	0.60 ± 0.03a	15.75 ± 0.69a	21.02 ± 0.43a	40.05 ± 1.22a	1.55 ± 1.02a	86.66 ± 2.42a	3179.11 ± 51.72a	13.13 ± 1.34a
	2	2.62 ± 0.41ab	0.61 ± 0.02a	14.99 ± 0.37b	25.73 ± 0.15b	36.78 ± 0.49b	6.91 ± 1.49b	98.56 ± 2.37b	3470.09 ± 31.16b	12.28 ± 0.70a
	3	2.76 ± 0.35ab	0.56 ± 0.04a	14.32 ± 0.42c	28.17 ± 0.65c	35.46 ± 1.32b	9.90 ± 1.27c	106.19 ± 3.28c	3691.82 ± 62.13c	13.23 ± 0.14a
	4	2.39 ± 0.29b	0.58 ± 0.03a	14.08 ± 0.34c	31.83 ± 0.96d	34.93 ± 0.46b	13.27 ± 1.19d	117.47 ± 2.34d	3891.71 ± 74.31d	14.89 ± 0.23b
120 °	1	2.83 ± 0.27a	0.58 ± 0.04a	14.13 ± 0.51a	22.82 ± 1.10a	38.00 ± 0.77a	1.97 ± 0.86a	90.87 ± 1.49a	3195.79 ± 57.18a	12.48 ± 0.89a
	2	2.51 ± 0.19ab	0.55 ± 0.03a	13.91 ± 0.75a	28.72 ± 0.77b	39.26 ± 1.13a	3.67 ± 1.30b	99.9 ± 2.23b	3533.89 ± 43.91b	13.61 ± 0.79ab
	3	2.45 ± 0.31ab	0.54 ± 0.05a	13.91 ± 0.73a	33.49 ± 1.78c	38.72 ± 1.29a	6.68 ± 1.54b	112.28 ± 3.35c	3624.33 ± 24.56c	13.82 ± 0.38ab
	4	2.21 ± 0.33b	0.56 ± 0.03a	15.89 ± 1.20b	35.46 ± 0.93c	37.64 ± 2.09a	14.64 ± 1.32d	121.22 ± 4.64d	3972.35 ± 69.88d	14.20 ± 0.53b

1 Data are presented as mean values ± standard deviation. 2 The different lowercase letters a-d represents significant difference in the same column (* *p* < 0.05).

**Table 3:** Effect of deep-frying time on color parameters, hardness, and fracturability of fried dough twist (FDT) in different an infrared-assisted condition.

Infrared-Assisted Condition	Deep-Frying Time (min)	a *	b *	L *	*Δ*E *	BI	Hardness (g)	Fracturability (N)
100 °C for 3 min	3	14.19 ± 0.60a	23.73 ± 0.64a	40.78 ± 0.68a	-	89.17 ± 3.12a	2914.25 ± 60.74a	12.56 ± 0.22a
	4	14.63 ± 1.32a	25.85 ± 0.58b	34.78 ± 0.72b	6.38 ± 1.35a	102.23 ± 1.32b	3179.11 ± 51.72b	13.13 ± 0.24ab
	5	14.32 ± 0.42a	28.17 ± 0.65c	35.46 ± 1.32b	6.93 ± 1.17a	106.19 ± 3.28b	3691.82 ± 62.13c	13.23 ± 0.14b
120 °C for 2 min	3	13.08 ± 0.83a	23.89 ± 0.42a	42.48 ± 1.65a	2.04 ± 1.52b	87.09 ± 2.34a	3325.73 ± 38.19a	12.45 ± 0.59a
	4	14.03 ± 1.57a	26.8 ± 0.51b	38.12 ± 0.91b	4.07 ± 1.37a	98.04 ± 2.19b	3470.09 ± 31.16b	12.28 ± 0.70ab
	5	13.91 ± 0.75a	28.72 ± 0.77c	39.26 ± 1.13b	5.22 ± 1.28a	99.9 ± 2.23b	3533.89 ± 43.91c	13.61 ± 0.79b

1 Data are presented as mean values ± standard deviation. 2 The different lowercase letters a–c represents significant difference in the same column (* *p* < 0.05).

**Table 4:** Moisture content, water activity, color parameter, texture parameters, acrylamide content, 5-(hydroxymethyl)-2-furfural (HMF) content, and oil content in the deep-frying and infrared pretreatment deep-frying fried dough twist (FDT).

	Deep-Frying (Sucrose)	Deep-Frying (Maltitol)	Infrared-Assisted Deep-Frying (Maltitol)
Moisture content (%)	3.05 ± 0.26a	3.18 ± 0.23a	2.73 ± 0.29b
Water activity	0.61 ± 0.01a	0.64 ± 0.02a	0.59 ± 0.05a
a *	16.37 ± 0.14a	15.31 ± 1.18a	14.63 ± 1.32a
b *	29.05 ± 1.05a	20.41 ± 2.26a	25.85 ± 0.58c
L *	35.69 ± 3.16a	26.78 ± 2.14b	34.78 ± 0.72a
*Δ*E *	-	17.68 ± 1.78a	30.18 ± 2.28b
BI	109.00 ± 1.64a	106.72 ± 1.48a	102.23 ± 1.32b
Hardness (g)	2852.63 ± 22.51a	2922.5 ± 27.19b	3179.11 ± 51.72c
Fracturability (N)	12.89 ± 0.28a	12.38 ± 1.23ab	13.13 ± 0.24b
Oil content (%)	21.1 ± 1.4a	20.6 ± 0.8a	15.3 ± 0.5b
Acrylamide (μg/kg)	206.1 ± 87.49a	85.68 ± 13.38b	78.53 ± 13.52c
HMF (μg/kg)	1054.19 ± 29.14a	474.08 ± 26.16b	380.32 ± 23.36c

1 Data are presented as mean values ± standard deviation. 2 The different lowercase letters a-c represents significant difference in the same row (* *p* < 0.05).

## Data Availability

No new data were created or analyzed in this study. Data sharing is not applicable to this article.
